# Knowledge mapping and research trends of exosomes in pancreatic cancer: a bibliometric analysis and review (2013-2023)

**DOI:** 10.3389/fonc.2024.1362436

**Published:** 2024-04-24

**Authors:** Yongjiang Zhou, Jiajie Feng, Qingqing Wang, Yiwen Zhao, Hanyu Ding, Kexin Jiang, Hua Ji, Zheng Tang, Ruiwu Dai

**Affiliations:** ^1^ General Surgery Center, General Hospital of Western Theater Command, Chengdu, Sichuan, China; ^2^ Department of General Surgery, Affiliated Hospital of Southwest Medical University, Luzhou, Sichuan, China; ^3^ College of Medicine, Southwest Jiaotong University, Chengdu, Sichuan, China; ^4^ Pancreatic Injury and Repair Key Laboratory of Sichuan Province, General Hospital of Western Theater Command, Chengdu, Sichuan, China

**Keywords:** pancreatic cancer, exosomes, bibliometric study, research hotspots, CiteSpace, VOS viewer

## Abstract

**Objective:**

This review aims to provide a quantitative and qualitative bibliometric analysis of literature from 2013 to 2023 on the role of exosomes in PC, with the goal of identifying current trends and predicting future hotspots.

**Methods:**

We retrieved relevant publications concerning exosomes in PC, published between 2013 and 2023, from the Web of Science Core Collection. Bibliometric analyses were conducted using VOSviewer(1.6.19), CiteSpace(6.2.R4), and Microsoft Excel (2019).

**Results:**

A total of 624 papers were analyzed, authored by 4017 researchers from 55 countries/regions and 855 institutions, published in 258 academic journals. China (n=285, 34.42%) and the United States (n=183, 24.87%) were the most frequent contributors and collaborated closely. However, publications from China had a relatively low average number of citations (41.45 times per paper). The output of Shanghai Jiao Tong University ranked first, with 28 papers (accounting for 4.5% of the total publications). *Cancers* (n=31, 4.9%); published the most papers in this field. Researcher Margot Zoeller published the most papers (n=12) on this topic. Research hotspots mainly focused on the mechanisms of exosomes in PC onset and progression, the role of exosomes in PC early diagnosis and prognosis, exosomes promote the development of PC chemoresistance, and potential applications of exosomes as drug carriers for PC therapies. We observed a shift in research trends, from mechanistic studies toward clinical trials, suggesting that clinical applications will be the focus of future attention. Emerging topics were pancreatic stellate cells, diagnostic biomarkers, mesenchymal stem cells, extracellular vesicles.

**Conclusion:**

Our scientometric and visual analysis provides a comprehensive overview of the literature on the role of exosomes in PC published during 2013–2023. This review identifies the frontiers and future directions in this area over the past decade, and is expected to provide a useful reference for researchers in this field.

## 1 Introduction

Pancreatic cancer (PC) is still one of the most aggressive and fatal digestive system tumors, which has become the fourth leading cause of cancer-related death in the world with an overall 5-year survival rate of 8%–9% (1). The insidious onset of PC often makes early diagnosis challenging; over 80% of cases are discovered at an advanced stage when surgical resection has become unfeasible due to local infiltration or distant metastasis (2). In the past 10 years, even the most advanced diagnostic techniques, perioperative management and systemic anti-tumor therapy for advanced diseases have been applied, but the prognosis of patients has not improved significantly (3). Consequently, understanding the pathogenesis of PC and developing strategies for its early diagnosis and effective treatment have gained increasing attention.

Exosomes are 30-150 nm small vesicles secreted by most cells into the extracellular space. Under both physiological and pathological conditions, virtually all human body cells release exosomes into various bodily fluids including plasma, urine, saliva, bile and ascites (4, 5). Exosomes carry a variety of biologically active substances, such as nucleic acids, proteins, lipids and enzymes. These material act on adjacent target cells through autocrine or paracrine, or on specific distant target cells through fluid transport (4, 5). Moreover, exosomes directly and specifically bind to target cells through membrane fusion or endocytosis, showing complex functions in intercellular communication and compound exchange, and playing an important role in the occurrence of diseases (6). In recent years, exosomes have become a hot research field of PC. An increasing body of evidence suggests that exosomes can promote the proliferation, metastasis and angiogenesis of pancreatic cancer cells(PCCs), and also show great potential value in the early diagnosis, treatment and other clinical applications of PC (7). For example, engineered exosomes for targeted delivery of drugs and various miRs lead to better tumor regression than intact exosome applications (8, 9). Therefore, it is particularly important to reveal the role of exosomes in PC, which can lead to a better orientation of researchers toward early diagnosis and new treatment of PC based on exosomes. Over the past decade, hundreds of research articles on exosomes in PC have been published. Therefore, there is an urgent need to collect and analyze a large amount of literature on this topic as a reference for future research.

Bibliometrics is a crucial instrument for fully understanding the research status of specific scientific topics, employing mathematical and statistical techniques to quantify datasets obtained from citation indexes (10–12). It evaluates the characteristics, development trends, and research focal points of publications in a specific field from both quantitative and qualitative perspectives (13, 14). The purpose is to deeply understand the research status of specific fields, research hotspots and predict future research trends (15–17). In recent years, bibliometric has also been applied to immunotherapy, autophagy therapy, neoadjuvant therapy, and tumor microenvironment of PC (18–21), but no bibliometric research or visual analysis of exosomes in PC has been reported. By searching the Web of Science Core Collection (WOSCC), a comprehensive bibliometric analysis was conducted on the literature related to exosome in PC from 2013 to 2023. We used CiteSpace, VOSviewer and the scientometric online platform to conduct measure and visualize analysis the number of publications, countries/regions, institutions, authors, journal and keywords in the literature related to exosomes in PC, and summarized the research hotspots and development trends in this field.

## 2 Materials and methods

### 2.1 Data source and search strategy

We conducted a comprehensive literature search of the WoSCC database on August 31, 2023. Primary search terms were “pancreatic cancer”, “pancreatic carcinoma”, “PDAC”, and “exosome”. The detailed search strategy is provided in **Supplementary File S1**. The inclusion criteria were as follows: (1) Articles published from January 1, 2013 to August 31, 2023; (2) publications that focused on the theme of exosomes in PC; (3) original research or review articles; and (4) studies that were written in English. The exclusion criteria were as follows: (1) articles published before January 1, 2013; (2) publications that were not related to exosomes in PC; (3) early access, meeting abstracts, book chapters, editorials, letters, news articles, and (4) duplicate papers. Two authors (ZYJ and FJJ) read the titles and abstracts of the articles to determine whether to include or exclude them; in case of uncertainty, the full text was downloaded to perform a more detailed evaluation, and papers unrelated to exosomes in PC were excluded. Differences in judgment were resolved through discussion or by consulting experienced correspondents (DRW). ZYJ and FJJ extracted titles; information on countries, institutions, journals, and authors; keywords; and references from all eligible literature. All information was exported with the record content of “Full Record and Cited References,” and downloaded in plain text format.

### 2.2 Data analysis and visualization

For bibliometric analysis, several software tools are commonly used, including VOSviewer, CiteSpace, and Microsoft Office Excel (22). This study utilized the aforementioned three software tools and one online website (https://bibliometric.com/) for the following analyses.

CiteSpace is a bibliometric and visualization analysis tool developed by Professor Chaomei Chen, is proficient at exploring research hotspots, evolutionary paths, knowledge structures, and emerging trends in a given field (23). Therefore, we used CiteSpace (6.2.R4) to analyze the included literature, including for performing co-citation analysis of references, dual-map overlay of citations, and analysis of keywords with the strongest citation bursts. CiteSpace is capable of generating visual graphs with nodes and lines, each graph holding different implications in various analytical methods. Nodes represent entries, with their size indicating frequency. The strength of collaboration is also depicted by the thickness of the connections between nodes—the thicker the connection, the greater the collaboration. Centrality is a measure of a node’s importance. Nodes with a centrality greater than 0.1 occupy key positions in connecting other nodes, with purple rings indicating central nodes. Burst detection can reveal emerging academic trends and new subjects as well as forecast leading-edge research directions and potential hotspots. Blue lines serve as timelines, with citation burst detection displayed as red segments on the blue timeline, indicating the start year, end year, and duration of the burst. Z-scores and F-scores were used to adjust or normalize citation data and identify major citation pathways in dual-map overlays. The basic parameter settings for CiteSpace were as follows: 1) Time slices: January 2013 to August 2023, with each slice spanning one year; 2) pruning (minimum spanning tree and pruning sliced networks); 3) the selection criteria were set to the top 50 levels for each slice, with other settings at default values.

In this study, VOSviewer software (version 1.6.19) was primarily used to analyze the collaboration networks among countries, institutions, authors, and keywords. In the map, a node represents an item (such as an author, journal, or keyword), with the node size indicating the number of publications, and the color representing different years. The thickness of the lines between nodes indicates the strength of co-citation and collaboration (24). For keyword analysis, both author keywords and keywords plus were analyzed. A minimum occurrence of 15 times was set for selected keywords to construct the keyword co-occurrence network.

**Figure 1:**
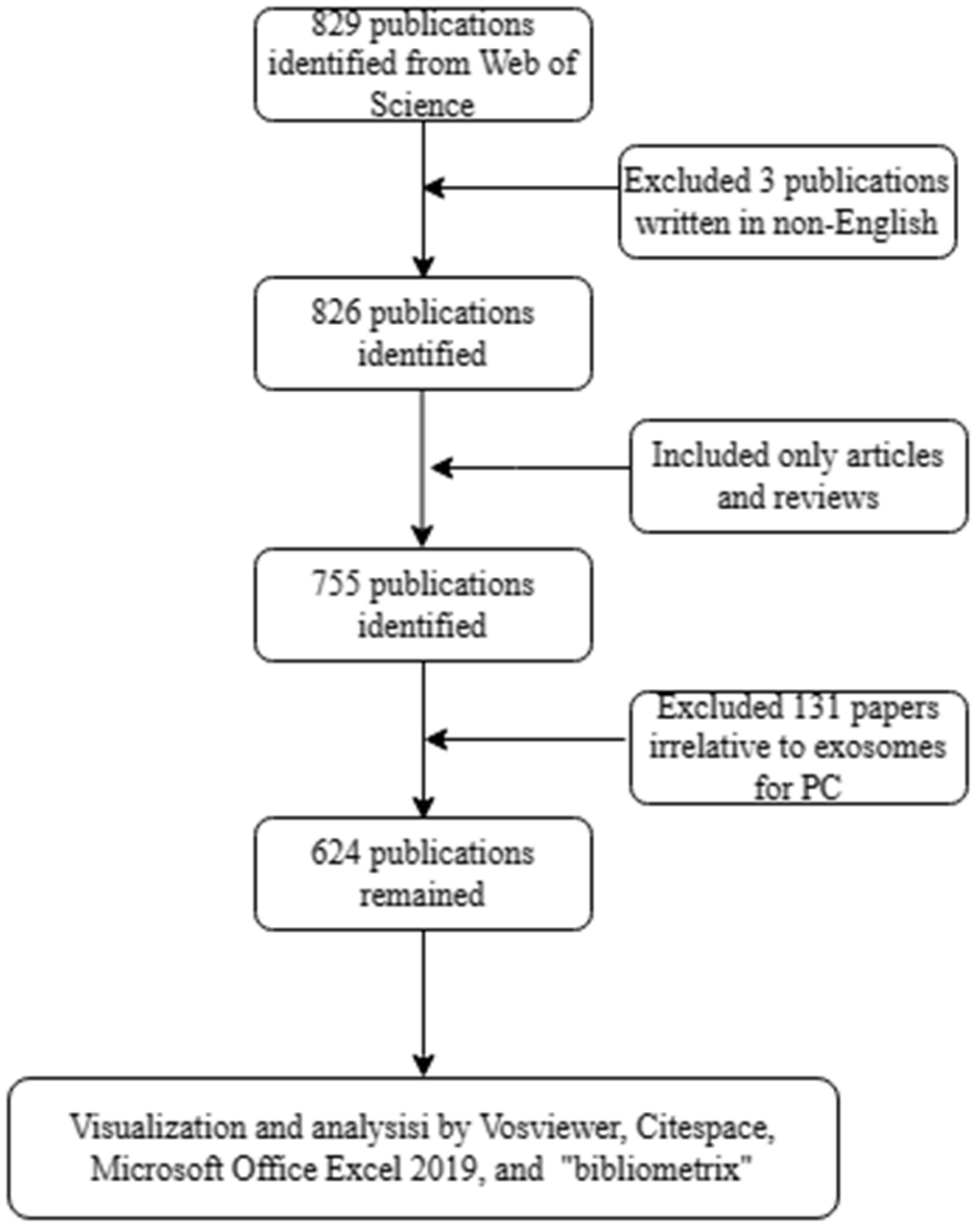
Flow diagram of the search strategy and analysis for research on exosomes in pancreatic cancer.

In addition, an international cooperation network has been established between countries using the scientific measurement online platform (https://bibliometric.com/). We also used Microsoft Office Excel 2019 (Microsoft, Redmond, Washington, USA) to build a polynomial regression model to predict the annual growth trend of publications. The data analysis flowchart is shown in **Figure 1**.

## 3 Results

### 3.1 Publication results and trends

**Figure 2:**
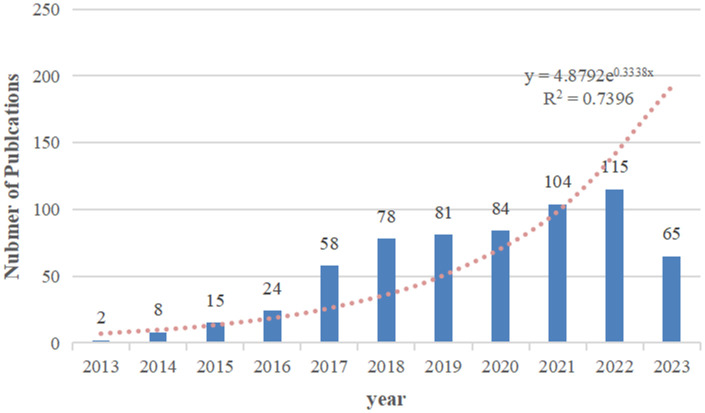
The number of annual research publications and growth trends on exosomes in pancreatic cancer from 2013 to 2023.

Based on our search strategy, a total of 624 publications on exosomes in PC were included in the bibliometric analysis, comprising 416 (66.7%) articles and 208 (33.3%) reviews. The annual number of publications on exosomes in PC from 2013 to 2023 is shown in **Figure 2**. Overall, the number of publications increased year by year, from 2 in 2013 to 115 in 2022. In addition, a total of 65 articles had been published as of August 31, 2023. A polynomial regression model was constructed to predict the number of annual publications (R^2 = ^0.7396). According to the fitting curve prediction, the number of publications on exosomes in PC will increase dramatically in the next few years. The above analysis indicates that the research interest in exosomes in PC has developed rapidly in the past decade, and will likely to continue to grow in the future.

### 3.2 Countries and organizations

**Figure 3:**
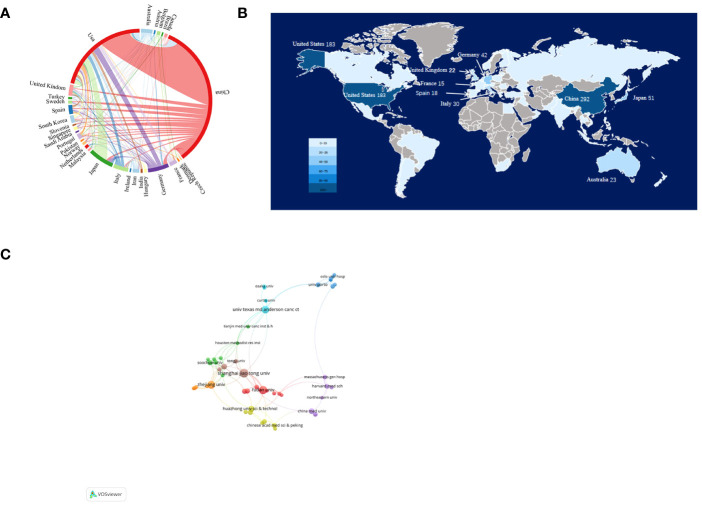
Articles related to the exosomes in pancreatic cancer published by country/region and institutions. **(A)** Collaboration between countries/regions based on https://bibliometric.com. **(B)** The publications density map of country/region. **(C)** Collaboration networks between institutions drawn by VOSviewer.

A total of 55 countries/regions and 855 institutions worldwide published publications related to exosomes in PC. China leads with the highest number of publications (292 publications, accounting for 34.97% of all publications worldwide; a total of 13,594 citations, with an average of 46.55 citations per paper and an H-index of 63). Following closely are the United States (US) (183 publications, accounting for 21.92%; a total of 17,875 citations, with an average of 97.68 citations per paper and an H-index of 66) and Japan (51 publications, accounting for 6.11%; a total of 2,060 citations, with an average of 40.39 citations per paper and an H-index of 22) (**Table 1**). Among the top 10 countries, China has the low average citations (46.55 times per paper). Although Spain published only 18 papers, it was cited an average of 213.17 times per paper, ranking first among the top 10 countries with the most published papers, followed by Germany (153.93 times), US (97.68 times), and France (65.33 times). In our research, we established collaborative network relationships between countries using a scientific measurement online platform at https://bibliometric.com/, revealing close cooperation between US and China with other countries/regions. (**Figures 3A, B**).

**Table 1:** The top 10 productive countries published literature related to exosomes in pancreatic cancer.

Rank	Country	Documents	Percentage	Total citations	Average citation	H-index
1	China	292	34.97%	13594	46.55	63
2	United States	183	21.92%	17875	97.68	66
3	Japan	51	6.11%	2060	40.39	22
4	Germany	42	5.03%	6465	153.93	25
5	Italy	30	3.59%	1157	38.57	22
6	Australia	23	2.75%	556	24.17	14
7	United Kingdom	22	2.63%	958	43.55	17
8	Spain	18	2.16%	3837	213.17	13
9	France	15	1.80%	980	65.33	11
10	South Korea	15	1.80%	484	32.27	11

The collaborative network of research institutions was constructed using VOSviewer (**Figure 3C**). Only the institutions with a minimum of five publications were included and 60 institutions were subsequently analyzed. The collaboration between institutions was a little closer than that between countries based on the total link strength. The top 12 institutions for the number of papers were listed in **Table 2**. Among them, 10 most productive institutions were located in China, followed by US (two institutions). Notably, the University of Texas M.D. Anderson Cancer Center leads both in terms of total citations and average citations per paper.

**Table 2:** The top 12 productive organizations in the field of exosomes in pancreatic cancer.

Rank	Organization	Country	Publications	Total citations	Average citations
1	Shanghai Jiao Tong Univ	China	28	1328	47.43
2	Fudan Univ	China	23	1156	50.26
3	Zhejiang Univ	China	22	1373	62.41
4	Univ Texas Md Anderson Cancer Center	United States	21	6235	296.90
5	Huazhong Univ Sci & Technol	China	13	287	22.08
5	China Med Univ	China	12	279	23.25
6	Nanjing Med Univ	China	12	496	41.33
7	Nantong Univ	China	12	401	33.42
8	Capital Med Univ	China	10	484	48.40

### 3.3 Analysis of authors

**Figure 4:**
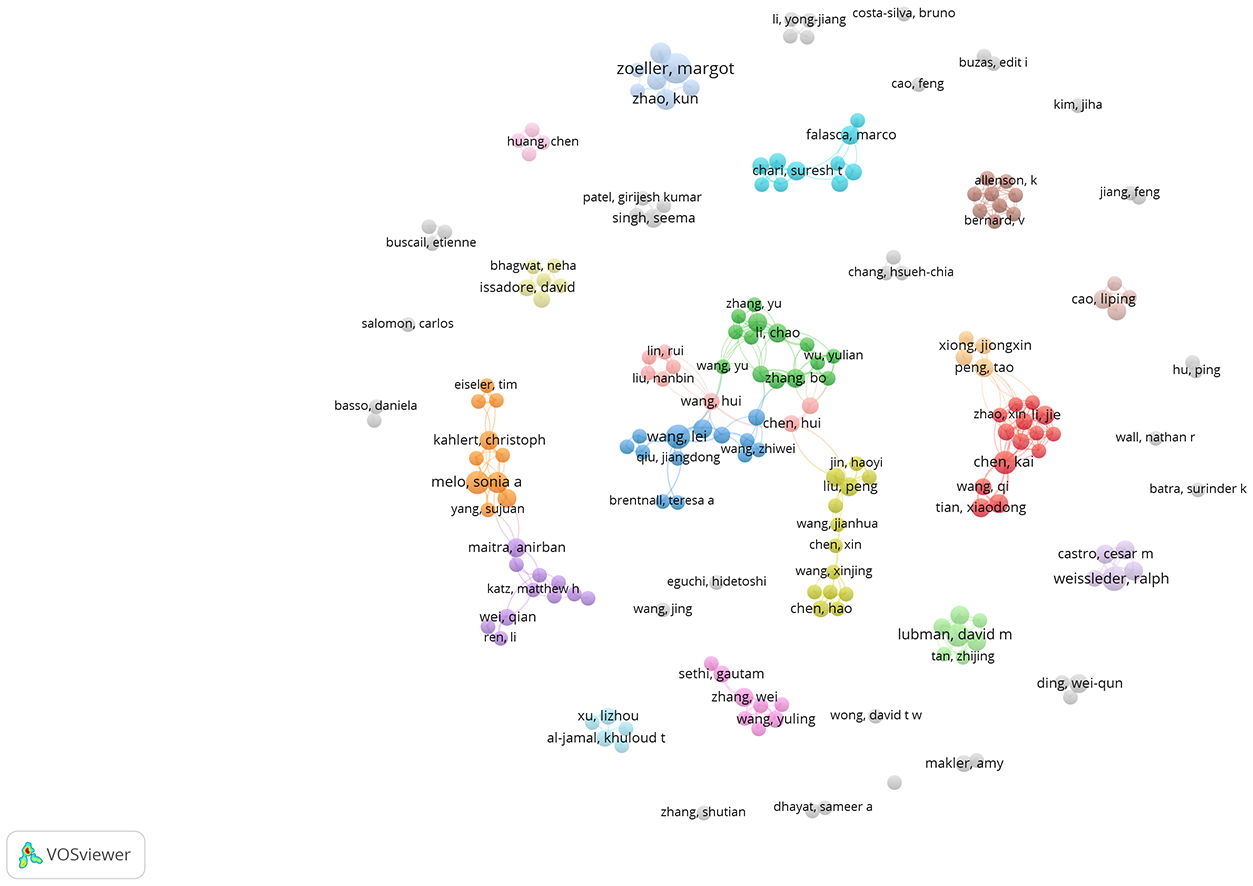
The co-authorship network analysis of authors. The size of the circle represents the number of publications; the thickness of the line represent collaborations between authors; the circle colors represents different clusters.

A total of 35 of 4017 authors had published at least 5 papers in the field of exosomes in PC. The top 10 authors with the most publications, four were from the US, and the other six were from China(n=3), Germany(n=2), and Portugal(n=1), respectively (**Table 3**). By the number of papers, Margot Zeoller published the most papers (n=12), followed by Wang Lei and Ralph Weissleder (n=8, respectively). Papers published by Raghu Kalluri who comes from Texas A & M Health Science Center had the highest total number of citations (4601 times). The cooperation networks analysis of authors who had published with a minimum of three publications were presented by the VOSviewer software (**Figure 4**). Authors within the same cluster/color, such as Melo, Sonia A and Kahlert Christoph, demonstrated strong collaborations. However, there was a noticeable lack of interaction between different cluster/color, indicating limited cooperation among distinct research groups.

**Table 3:** The top 10 authors who have contributed the most publications on exosomes in pancreatic cancer.

Rank	Author	Publications	Total citations	Country	Affiliation
1	Zoeller Margot	12	681	Germany	Heidelberg Univ
2	Wang Lei	8	239	China	Shandong Univ
3	Weissleder Ralph	8	1448	United States	Harvard Med Sch
4	Chen Kai	7	828	China	Peking Univ
5	Lubman David M	7	291	United States	Univ Michigan
6	Melo, Sonia A	7	4204	Portugal	Univ Porto Ipatimup
7	Kalluri Raghu	6	4601	United States	Texas A&M Hlth Sci Ctr
8	Wang Zhe	6	183	China	Shanghai Jiao Tong Univ
9	Zhao Kun	6	158	Germany	Heidelberg Univ
10	Lebleu Yalerie S	5	3895	United States	Texas A&M Hlth Sci Ctr

### 3.4 Analysis of journals

**Figure 5:**
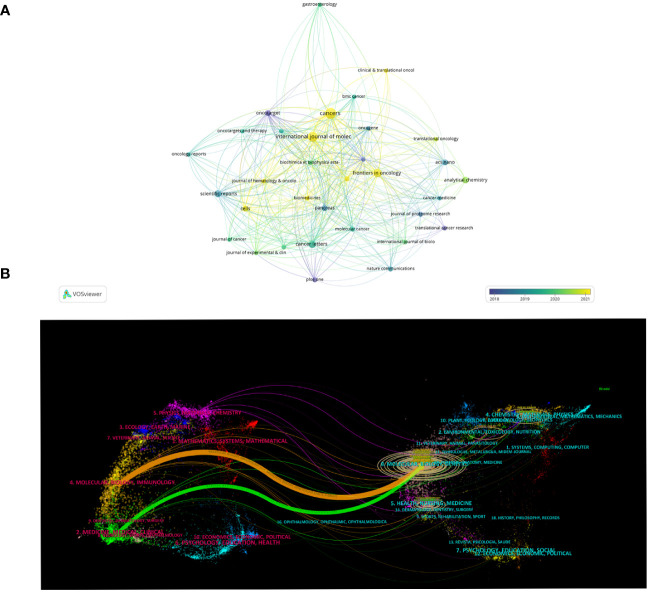
Journals related to exosomes in pancreatic cancer. **(A)** The overlay visualization map of journals associated with research on exosomes in pancreatic cancer. The circle size means the number of publications; the circle colors mean the average published year. **(B)** The dual-map overlay of journals related to the exosomes in pancreatic cancer.

A total of 258 journals have published the 624 publications concerning exosomes in PC. The top 10 journals published 146 papers, accounting for 23.03% of all publications (**Table 4**). *Cancers* published the most papers with 31 papers, followed by *International Journal of Molecular Sciences* (24), *Frontiers in Oncology* (18), *Cancer Letters* (14), *Scientific Reports* (12), *Analytical Chemistry* (11). Although *ACS Nano* has only published 9 articles, it has the highest total citations (1363) and average citations (151.44). *Cancer Letters* (825, 58.93), *Scientific Report* (560, 46.67), *Oncotarget* (662, 73.56) also have a higher number of total citations and average citations. In addition, the VOSviewer software was used to perform a visual overlap analysis for journals (n=33) that published at least 5 papers (**Figure 5A**). *Cancers, Frontiers in Oncolgy, International Journal of Molecular* are emerging journals related to exosome in PC in recent years.

**Table 4:** The top 10 productive journals associated with exosomes in pancreatic cancer.

Rank	Source	Documents	Citations	Average Citations	Country
1	Cancers	31	641	20.68	Switzerland
2	International Journal of Molecular Sciences	24	574	23.92	Switzerland
3	Frontiers in Oncology	18	127	7.06	Switzerland
4	Cancer Letters	14	825	58.93	Ireland
5	Scientific Reports	12	560	46.67	United Kingdom
6	Analytical Chemistry	11	393	35.73	United States
7	Acs Nano	9	1363	151.44	USA
8	Cells	9	171	19.00	Switzerland
9	Oncotarget	9	662	73.56	USA
10	Pancreas	9	192	21.33	USA

### 3.5 Dual-map overlays of the exosomes in PC

The dual-mapping overlay reveals the overall scientific contributions. As is shown in **Figure 5B**, the left side represents the citing journal, and the right side represents the cited journal. The curve between the two sides represents the reference relationship, indicating the flow of knowledge and the relationship between different research areas. And we found that the orange paths were the main citation paths, which indicating that publications from molecular, biology, genetics were frequently cited by publications from molecular, biology, immunology (z=5.64, f=26348).

### 3.6 Analysis of references

**Figure 6:**
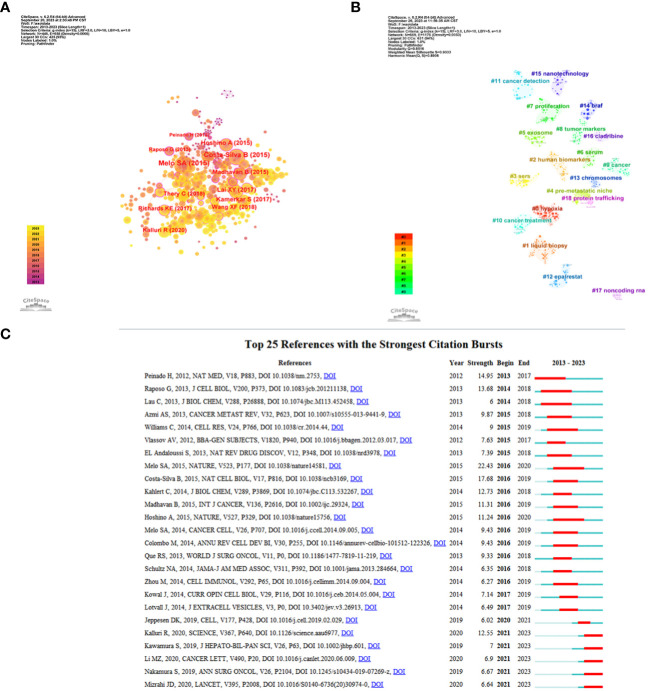
References related to the exosomes in pancreatic cancer. **(A)** Clustering analysis of the exosomes in pancreatic cancer cocitation network drawn by CiteSpace. **(B)** Cluster view of co-citation references related to exosomes in pancreatic cancer visualized by CiteSpace. **(C)** The top 25 with the strongest citation bursts based on analysis in CiteSpace.

The top 10 highly co-cited references for studies on exosomes in PC are shown in **Table 5**. Co-citation was first proposed by the American information scientist Small in 1973 for measuring the relationship between publications, as an indicator of the significance of a publication’s impact on a specific field. Here, CiteSpace was used for visualization network analysis of co-cited references, and the top N was set as 50 to perform a co-cited analysis of the publications (**Figure 6A**). Further cluster analysis was performed on the publications with the greatest impact (**Figure 6B**). In addition, we identified the top 25 references with the strongest citation bursts, which indicate that they have been widely cited by other studies and received particular attention in a certain period. **Figure 6C** illustrates that since 2012, the publication with the strongest citation bursts was authored by Melo SA et al. (25), which was published in *Nature* in 2015. This was closely followed by Costa-Silva B et al.’s work (26) published in *Nature Cell Biology* also in 2015, and then by Peniado H et al.’s paper (27) featured in *Nature Medicine* in 2012.

**Table 5:** The top 10 highly co-cited references of exosomes in pancreatic cancer.

Rank	Title	First Authors	Journal	Type	Year	Total Citations
1	Glypican-1 identifies cancer exosomes and detects early pancreatic cancer	Melo SA	Nature	Article	2015	145
2	Pancreatic cancer exosomes initiate pre-metastatic niche formation in the liver	Costa-Silva B	Nature Cell Biology	Article	2015	112
3	Exosomes facilitate therapeutic targeting of oncogenic KRAS in pancreatic cancer	Kamerkar S	Nature	Article	2017	79
4	A microRNA signature in circulating exosomes is superior to exosomal glypican-1 levels for diagnosing pancreatic cancer	Xianyin Lai	Cancer Letters	Article	2017	75
5	Tumour exosome integrins determine organotropic metastasis	Ayuko Hoshino	Nature	Article	2015	74
6	Combined evaluation of a panel of protein and miRNA serum-exosome biomarkers for pancreatic cancer diagnosis increases sensitivity and specificity	Bindhu Madhavan	Int J Cancer	Article	2015	71
7	The biology, function, and biomedical applications of exosomes	Raghu Kalluri	Science	Review	2020	60
8	Minimal information for studies of extracellular vesicles 2018 (MISEV2018): a position statement of the International Society for Extracellular Vesicles and update of the MISEV2014 guidelines	Thery Clotilde	Journal of Extracellular Vesicles	Article	2018	59
9	Cancer-associated fibroblast exosomes regulate survival and proliferation of pancreatic cancer cells	Richards K. E.	Oncogene	Article	2017	58
10	Hypoxic Tumor-Derived Exosomal miR-301a Mediates M2 Macrophage Polarization via PTEN/PI3K gamma to Promote Pancreatic Cancer Metastasis	Wang Xiaofeng	Cancer Research	Article	2018	58

### 3.7 Keyword co-occurrence cluster analysis

**Figure 7:**
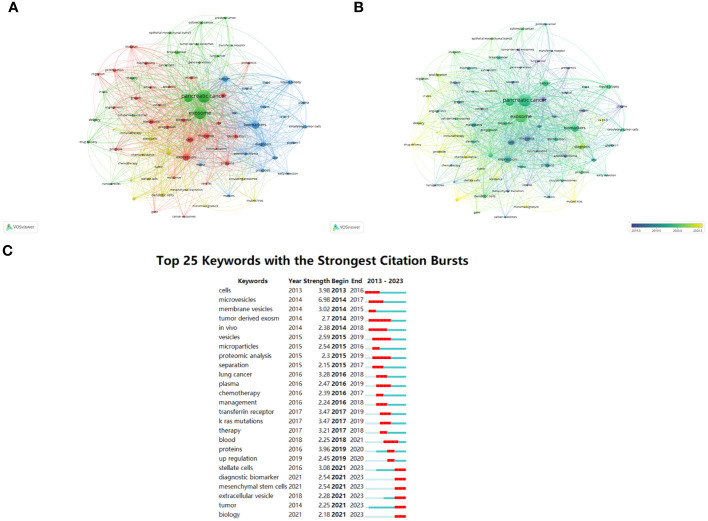
Keywords related to exosomes in pancreatic cancer. **(A)** Network visualization of keywords drawn by VOSviewer. The circle size means the keywords frequency of occurrence; the circle colors mean different clusters; **(B)** Overlay visualization of keywords drawn by VOSviewer. The circle size means the keywords frequency of occurrence; the circle colors mean the average published year. **(C)** The top 25 keywords with the strongest citation bursts drawn by CiteSpace. A red bar indicates high citations in that year.

VOSviewer software’s keyword co-occurrence analysis visually shows that a total of 2291 keywords were extracted. **Table 6** illustrates the high-occurrence keywords from articles on exosomes in PC from 2013 to 2023. Keywords that appeared more than 15 times were used to build a visual network of keyword clusters; 78 keywords met the threshold analysis. The network was divided into four clusters with different colors; we summarized these four clusters as Cluster 1 (red), mainly focused on mechanistic research on exosomes in PC occurrence and progression; Cluster 2 (blue), which includes the role of exosomes in early diagnosis and prognostic evaluation of PC; Cluster 3 (yellow), focused on exosome-mediated PC resistance; and Cluster 4 (green), centered on the application of exosomes as drug delivery carriers in PC (**Figures 7A, B**). We also used CiteSpace to identify the top 25 keywords with the strongest citation burst. Keyword burst analysis was used to reflect the stage hotspots and trends in research on exosomes in PC (**Figure 7C**). Microvesicles, cell, and proteins were the three strongest keywords. The keywords that identify the research frontiers and continue to 2023 are: pancreatic stellate cells, diagnostic biomarkers, mesenchymal stem cells, extracellular vesicles.

**Table 6:** Top 20 keywords of documents on exosomes in pancreatic cancer.

Rank	keyword	occurrences	Rank	keyword	occurrences
1	pancreatic cancer	451	11	liquid biopsy	64
2	exosome	430	12	microvesicles	59
3	extracellular vesicles	213	13	proteins	58
4	biomarkers	169	14	gemcitabine	57
5	expression	137	15	dendritic cells	54
6	cancer	107	16	breast cancer	47
7	cells	100	17	identification	44
8	microrna	94	18	biogenesis	42
9	diagnosis	83	19	progression	41
10	metastasis	74	20	growth	41

## 4 Discussion

### 4.1 General information

In this study, we used VOSviewer and CiteSpace software to perform a bibliometric and visualization analysis of 624 papers based one the literature related to exosomes in PC published between January 1, 2013 and August 31, 2023. This study examines the current status of global research on exosomes in PC, summarize research hotspots, and predict future research trends. Our results suggest that this research field will continue to grow, with the potential for enhancing our understanding of exosomes in PC research in the future.

The change in the number of scholarly publications serves as a crucial indicator for gauging the trajectory of progress within a particular field. Over the past 10 years, publications related to exosomes in PC have shown a significant increasing tendency. In 2013, only two articles were published; however, the number of publications has increased year by year since, and the annual number of publications has exceeded 50 since 2017. As of 2022, 115 articles were published. These results suggest that field of research examining the role of exosomes in PC has begun to develop rapidly in the past 10 years, and, in the future, more articles in this field will be published.

The number of publications on a field of research from a given country is an important indicator of the country’s interest in that field. **Table 1** shows the number of publications from the top 10 countries. US had the highest number of total citations (n =17875) and H-index (H=66), which indicated that the US has made great contributions in this field and is in a leading position. Although Spain has only 18 publications, it has the highest average citations (n=213.17), indicating the overall high quality of its papers. In addition, China has the most publications (n=292), but its average citations are low (n=46.55), suggesting that Chinese scholars need to publish high-quality papers to improve their academic standing. The US and China, which published the most papers, have made significant contributions to the study of exosomes in PC. However, the number of references and the H-index of studies cited by American scholars are noticeably higher than those of Chinese scholars, indicating that the US maintains a higher level of research output, while China is an emerging major power. Academic capability is primarily contingent upon a nation’s economic conditions and its government’s research expenditure (28). The high economic status and substantial healthcare spending of the US may partially account for its vast scientific output and strong academic influence (24, 28). Despite China’s rapid development and dominant position in this field, the relatively lower quality of many papers indicates a need for strengthened regional cooperation and academic influence. To enhance China’s quality of research and academic influence, certain measures need to be taken. First, it is necessary to increase economic investment to ensure sufficient resources for conducting high-quality research, in terms of both depth and breadth; second, healthcare investment should also be intensified to better support scientific exploration and practice within relevant fields. **Figure 3A** shows the partnerships between the top 30 countries with the most publications—the US had the closest cooperation with other countries/regions. The US, along with Asian countries such as China and Japan, as well as European countries like Germany, Italy, and the United Kingdom, continued to play a crucial role in national cooperation. However, cooperation from other developing countries remained relatively limited. It is imperative for these nations to enhance their collaboration efforts in order to foster global advancements in the field of research on exosomes in PC.

In terms of institutions, Shanghai Jiao Tong University published 28 papers, covering the roles of exosomes in PC proliferation and metastasis (29–31), drug resistance and immune escape (32, 33), early diagnosis and prognosis (34–36), and treatment among other aspects (32, 37), making it the institution with the highest number of publications in this area. The most popular of these publications was a randomized controlled trial conducted by Zhengjun Qiu, which reported the purification of exosomal miR-301a-3p from hypoxic PC cell supernatants and explored the role of exosomes, and the potential mechanisms involved, in PC progression. The results of this study suggest that exosomal miR-301a-3p derived from hypoxic PC cells induces macrophage M2 polarization through the activation of the PTEN/PI3Kγ pathway, promoting PCCs migration, metastasis, and epithelial-mesenchymal transition (EMT) (33). Furthermore, another article by these authors reporting that M2 macrophage-derived exosomes promote PC angiogenesis by targeting E2F2 has also garnered significant attention (38), potentially offering diagnostic and therapeutic strategies against angiogenesis in PC. Their contributions have not only significantly advanced the development of research on the role of exosomes in PC, but also played a crucial role in shaping current understanding of the therapeutic potential of exosomes in cancer treatment. **Figure 3C** shows institutional partnerships with more than five publications; although some institutions had cooperated with other national institutions, such as Shanghai Jiao Tong University and UT MD Anderson Cancer Center, they were found to mainly cooperate with domestic institutions such as Fudan University, Zhejiang University, and China Medicine University. It was found that in the field of research on the role of exosomes in PC, there is limited collaboration and sharing of findings among institutions across different countries. Moreover, most collaborative efforts were restricted to domestic institutions. Given the increasing utilization of exosomes in oncology and other medical domains, as well as the deepening of research in this area, this limited cooperation has significantly impeded the progress of related research fields. Consequently, it is crucial to enhance international institutional cooperation and collectively drive the advancement of research in this field.

Margot Zoeller from Germany published the most publications (12,1.89%), followed by Wang Lei et al. (8, 1.26%) from China and Ralph Weissleder et al. (8, 1.26%) from the US. These three authors have made great contributions to the field. As pioneers in this field, the Nobel Prize laureates Professor James E. Rothman and Randy W. Schkann from the US, and Professor Thomas C. Sudhof from Germany, have made outstanding contributions to our understanding of the vesicle transport regulation mechanism of exosomes (39), maintaining a high level of research in this field. Therefore, their contributions have played a crucial role in promoting our understanding and therapeutic application of exosomes.

As an emerging method for the early detection and treatment of PC, the study of exosomes has always been a hot topic in journals. *Cancers* published the most papers—with 31 papers published, this journal has made great contributions to the field. Although *ACS Nano* has only published nine articles, this journal has the highest total (1363) and average citations (151.44), which indicates that the papers published in the journal were of high quality. The dual-map overlay shows that current research on the role of exosomes in PC is mainly concentrated in the fields of molecular biology and medicine. In recent years, with the deepening of research, various disciplines have gradually infiltrated this field. For example, Jina et al. explored the diagnostic value of exosomes in PC by using machine learning algorithms and nanofluidic techniques (40), while Daniel et al. proposed a method for PC diagnosis and treatment based on surface acoustic wave exosome lysis and ion-exchange nanomembrane detection of exosomal RNA (41). A multidisciplinary approach can better integrate resources and ideas to achieve novel scientific breakthroughs and develop comprehensive solutions to global problems. Most of the top ten highly cited papers were published before 2017, as more citations can be found over time. However, the paper “The biology, function, and biomedical applications of exosomes”, published by Kalluri and his team in Science in 2020, represented a milestone in the study of exosomes (6). This article systematically reviews the role of exosomes in the diagnosis, treatment, and prognosis of diseases.

### 4.2 Clinical research

In order to investigate global research trends and hotspots in the field of exosomes in PC, we utilized VOSviewer to conduct a co-occurrence analysis of all related keywords. This study incorporated 2291 high-frequency keywords associated with exosomes in PC from 2013 to 2023. The resultant keyword co-occurrence network was divided into four distinct clusters, each represented by different colors corresponding to separate research topics. In the following sections, these four clusters will be analyzed further in detail.

#### 4.2.1 Effect of exosomes on proliferation, metastasis and angiogenesis in PC

A review and summary of relevant research shows that Cluster 1 (red) primarily focuses on investigating the mechanisms underlying the involvement of exosomes in PC occurrence and progression. Notably, recent research in this area has been particularly focused on understanding the roles of proliferation, metastasis, and angiogenesis. In 2013, Soo et al. published a groundbreaking article titled “Unexpected gain of function for the scaffolding protein plectin due to mislocalization in pancreatic cancer” (42), which is considered one of the most significant and influential contributions in this field. In their study, they utilized PDAC orthotopic mouse models to explore the role of exosome transport in the mislocalization of plectin to the surface of PDAC cells. Their findings revealed that this abnormal expression and mislocalization of plectins can drive the proliferation, metastasis, and angiogenesis of PDAC cells (42).

Exosomes play a key role in the proliferation of PCCs. Exosomes released by PCCs contain various signaling molecules—including miRNAs, mRNAs, lncRNAs and proteins—that promote cell proliferation and infiltration, contributing to the growth and spread of PC (43). Among these, miRNA is recognized as a pivotal molecular actor that orchestrates gene regulation post-transcriptionally (44). For instance, a recent study demonstrated that exosome-mediated delivery of miR-3960 inhibits TFAP2A, leading to increased proliferation and invasion of PCCs (45). Another study utilized exosomes from hucMSCs to deliver exogenous miR-145-5p, revealing that upregulation of miR-145-5p suppresses the proliferation and invasion of PDAC cells (via suppression of Smad-3/TGF-β-mediated EMT), promotes apoptosis, and induces cell cycle arrest. *In vivo* experiments further confirmed that miR-145-5p overexpression reduces tumor proliferation in a mouse model (46). These results demonstrate that exosome components, which include miRNAs, are a promising therapeutic tool for inhibiting the proliferation and invasion of PC.

Metastasis is a complex and multistep process involved in cancer progression. Various studies have reported that PC-derived exosomes play a role in promoting premetastatic niche formation and preparing sites for tumor cell invasion, colonization, and survival (47). Costa-Silva et al. discovered that PDAC-derived exosomes can promote liver premetastatic niches, potentially increasing liver metastasis (26). They also identified high expression of macrophage migration inhibitory factor in PDAC-derived exosomes, which, when blocked, prevents the formation of these niches and metastasis (26). Under hypoxic conditions, highly expressed exosomal miR-301a3p induces polarization of M2 macrophages by activating the PTEN/PI3Kg signaling pathway. This leads to accelerated invasion, migration, and metastasis of PC (33). Further studies found that M2 macrophage-derived exosome miR-501-3p promotes liver and lung metastasis by inhibiting *TGFBR3* expression through activation of the TGF-β signaling pathway (48). Additionally, numerous studies have highlighted the role of lncRNAs carried by exosomes in tumor invasion and metastasis. These lncRNAs can interact with miRNAs to form an invasion-metastasis cascade (49). For example, Li et al. discovered that highly expressed PDAC-derived exosomal lncRNA *Sox2ot* promotes EMT, thereby facilitating invasion and metastasis in PDAC (50). In summary, these findings demonstrate the involvement of exosomes in various processes related to PC invasion and metastasis.

Angiogenesis, a key process in the progression of advanced PC, leads to tumor vascularization and promotes distant metastasis (51). However, the underlying mechanisms are yet to be fully understood. Guo et al. indicated that hypoxic exosome UCA1 derived from PCCs is highly expressed and can stimulate cell migration and angiogenesis in HUVECs (52). In another study focusing on HMVECs targeted by PANC-1-derived exosomes (a PC cell line), it was found that miR-27a contained within these exosomes affected proliferation, invasion, and angiogenesis of HMVECs (53). Specifically, PC cell-derived exosomal miRNA-27a stimulated angiogenesis of HMVECs in PC by suppressing BTG2. These findings shed light on how specific components carried by exosomes contribute to the complex processes involved in PC progression.

Exosomes have been shown to act as a “double-edged sword” in cancer progression, promoting tumor proliferation, metastasis, and angiogenesis on the one hand, while also inhibiting cancer progression on the other. Therefore, exploring the functions and mechanisms of exosomes in PC development is an important research goal. In recent years, new potential regulatory factors, such as m(6)A modification regulators PDL-1, cfDNA, and ctDNA, have emerged as areas of interest. Investigation of whether these factors contribute to the proliferation and metastasis of PC through exosome-mediated signaling cascades has become a significant area of focus within this field.

#### 4.2.2 Exosomes can be used as biomarkers for early diagnosis and prognosis of PC

In Cluster 2 (blue), research primarily revolves around exploring the role of exosomes in the early diagnosis and prognostic evaluation of PC. Notably, several key keywords have emerged as recent research hot topics within this cluster, including miRNA, glypican-1, biomarkers, liquid biopsy, and prognosis. Overall, studies focusing on these areas aim to advance the understanding of how exosomes can serve as promising tools for early detection and prognostic evaluation in PC.

PC is difficult to diagnose early because of its vague clinical symptoms and signs. Imaging techniques such as computed tomography and endoscopic ultrasonography currently have limited effectiveness in detecting early-stage PC (54). Although the serum biomarker CA 19-9 is commonly used for PC diagnosis, it has moderate sensitivity (79%–81%) and specificity (82%–90%) (55). Therefore, relying solely on CA 19-9 can be challenging for confirming a PC diagnosis, especially in patients with non-specific symptoms. Exosome markers based on serum or body fluids show promise as non-invasive diagnostic methods, particularly in the early stages of PC. Exosomal miRNAs are stable and enriched in serum exosomes, offering a simple, accurate, and reliable method for early detection of certain malignant tumors (56). In an experiment, Xu et al. performed qRT-PCR analysis and found that plasma exosomes miR-196a and miR-1246 were highly enriched in PC exosomes. Furthermore, plasma exosomal miR-196a and miR-1246 levels in patients with localized PC were significantly higher than those in healthy subjects (57). It was also found that the levels of serum exosomes miR-17-5p and miR-21 were higher in PC patients than in non-PC patients and healthy participants (58). The sensitivity and specificity of miR-17-5p and miR-21 in the diagnosis of PC were 72.7% and 92.6%, and 95.5% and 81.5%, respectively. Further studies have found that high levels of miR-17-5p are significantly associated with the metastasis and advanced stages of PC, and may be useful as a serum biomarker for PC diagnosis (58). However, a large number of studies have shown that miR-21 is also highly expressed in other malignant tumors, including gastric cancer, ovarian cancer, breast cancer, colon cancer, and liver cancer (59–62). Lai and colleagues reported that high levels of exosomal miR-10b, miR-21, miR-30c, and miR-181a and low levels of exosomal miR-let7a easily distinguish PDAC from CP patients, compared with exosomal GPC1; furthermore, elevated exosomal miR levels were found to reduce to normal within 24 hours after PDAC resection. In addition, in their study, all 29 PDAC cases showed significantly elevated exosomal miR-10b and miR-30c levels, while CA 19-9 levels were normal or slightly elevated in eight cases (63). Therefore, evaluation of the levels of these miRNAs in serum is more useful than detection of serum exosomal GPC-1 or plasma CA 19-9 for distinguishing PaCa from CP. In addition to miRNAs, the future focus will be on other nucleic acids—such as mRNA (64, 65), lncRNA (50, 66), circular RNA (67, 68)—as exosomal components, to further explore minimally invasive biomarkers for predicting, diagnosing, and monitoring PC as well as identify new specific drug targets for the treatment of this form of cancer.

Glypicans, a group of cell-surface glycoproteins attached by GPI anchors, have shown potential in PC diagnosis (69). Glypican-1(GPC1) is highly expressed in PCCs and adjacent fibroblasts, but less expressed in the normal pancreas and in chronic pancreatitis (25). Mass spectrometry analyses have identified the specific enrichment of GPC1 on cancer cell-derived exosomes. Detection of GPC1+crExos in the serum of PC patients has demonstrated high specificity and sensitivity for distinguishing non-neoplastic pancreatic diseases from early and advanced PC (25). Additionally, lower levels of serum GPC1-Exo are associated with poor patient survival. However, recent studies indicate that plasma levels of GPC1+crExos are also elevated in stage II colorectal cancer, suggesting that GPC 1 + crExos may not be a specific marker for PC diagnosis (70). Furthermore, high expression of GPC-1-Exo has been observed in breast cancer tissues and cells relative to normal breast tissues (71). Further research is necessary to explore the potential application of GPC1 in diagnostic or therapeutic strategies for PC.

In summary, while exosome-mediated non-invasive diagnostic strategies show promise over traditional tumor markers such as CA19-9 for early detection of PC, there remains an urgent need to combine exosomal miRNA, DNA, and protein analysis with traditional serum tumor markers to improve the specificity and sensitivity of PDAC diagnosis.

Exosomes not only serve as potential markers for early diagnosis but also demonstrate prognostic evaluation capabilities in PC. High expression of miR-196b, identified through miRNA array analysis, has been found to be associated with poor prognosis in PC patients (72). Additionally, a study investigating the changes in GPC1 levels on serum exosomes before and after surgical resection observed a significant decrease post-surgery, suggesting that exosomes containing GPC1 could be used as a non-invasive prognostic marker (73). Furthermore, Kawamura et al. discovered that specific miRNAs, such as miR-4525, miR-451A, and miR-21, found in portal vein-derived exosomes may serve as biomarkers to identify patients at high risk of recurrence with poor postoperative outcomes (74). Although numerous studies have demonstrated the importance of exosomes in predicting prognosis, further research and clinical trials are necessary to establish their routine use as prognostic markers for PC.

#### 4.2.3 Exosomes promote the development of chemoresistance

Cluster 3 (yellow) focused on exosome-mediated PC resistance. A significant challenge in PC treatment arises from the development of drug resistance by cancer cells. Increasing evidence indicates that exosomes play a crucial role in chemoresistance (75). Currently, first-line chemotherapeutic options for advanced PC are primarily limited to FOLFIRINOX, modified FOLFIRINOX, and gemcitabine-based multi-drug combinations (76). However, chemoresistance, especially GEM resistance, severely reduces the benefits derived from these treatments (77). Notably, interactions between PCCs and other cells within the tumor microenvironment (TME) occur via exosomal RNA, proteins, and associated signaling pathways. These complex interactions contribute to multifactorial chemotherapeutic resistance that ultimately leads to poor clinical prognosis (78).

In the TME of PC, certain cells, such as macrophages, play a significant role in the development of drug resistance. Recent studies have shed light on how exosomes derived from M2-macrophages contribute to this process. For example, researchers discovered that miR-365 carried by M2-macrophage-derived exosomes enhances chemoresistance by activating deoxycytidine kinase. This enzyme metabolizes gemcitabine (GEM) into an inactive form (79). In addition, it has been shown that genetically modified Rab27 a/b-knockout mice are unable to secrete exosomes. These mice metabolize GEM more effectively than wild-type mice, and the treatment of PDAC-carrying mice with an miR-365 antagonist was found to restore sensitivity to GEM (79). Another study reported that lncRNA SBF2-AS1 carried by M2 macrophage-derived exosomes inhibits the expression of miR-122-5p. This inhibition leads to upregulation of X-linked inhibitor of apoptosis protein and subsequently reduces the efficacy of GEM (80). These insights suggest that targeting M2 macrophage-derived exosomes could provide a new direction for overcoming PC resistance. However, further research is needed to fully elucidate these mechanisms and develop effective therapeutic strategies based on them.

Numerous studies have indicated that cancer-associated fibroblasts (CAFs) in the TEM can induce chemotherapeutic resistance to GEM by secreting exosomal miRNAs (81). Fang et al. investigated the role of CAFs in regulating drug resistance through the transfer of exosomal miR-106b to cancer cells (82). Their findings revealed that CAF-derived exosomes possess inherent resistance to GEM when exposed to conditioned medium. Moreover, they found that treating these cancer cells with GEM further amplified the impact of either CAFs or their exosomes on PCCs proliferation as well as increased the levels of miR-106b. Interestingly, pretreatment of CAFs with a miR-106b inhibitor resulted in reduced expression of miR-106b within the released exosomes and subsequently decreased the resistance of cancer cells to GEM (82). In another study, researchers discovered an increase in exosome release from CAFs after exposure to GEM. These additional exosomes led to enhanced expression of chemoresistance-inducing factor Snail (SNAI1) within receptor epithelial cells (83). SNAI1 promotes PDAC cell proliferation and drug resistance by targeting miR-146a. Finally, blocking the secretion of CAF-derived exosomes using GW4869 significantly improved the efficacy of GEM against PCCs, while simultaneously reducing tumor volume. These results highlight the crucial role played by CAF-secreted exosomes in mediating GEM resistance (83).

Although numerous studies have investigated exosome-mediated resistance in PC, our understanding of how exosomes mediate and transfer chemotherapeutic resistance remains limited. Further research is required to identify strategies to overcome chemoresistance and develop improved treatment options for PC patients.

#### 4.2.4 Exosomes as drug carriers for PC therapy

Cluster 4 (green) centered on the application of drug carriers in PC. Exosomes, with their favorable biodistribution, biocompatibility, low immunogenicity, and ability to penetrate biofilms, have recently garnered extensive attention as potential vehicles for drug delivery in PC treatment (84). PC is often diagnosed at an advanced stage, with a low median survival rate. In addition to active chemotherapy and surgical resection, gene therapy provides doctors and patients with new treatment options, even in patients with locally advanced disease. Current research on exosome-based drug delivery in PC primarily focuses on loading genetic materials such as small interfering RNA (siRNA) and miRNA into exosomes to inhibit PC proliferation and metastasis (85). For example, KRAS mutations are prevalent within PC cases. Detailed mechanism analysis has revealed that this mutation often results in the replacement of the glycine residue at codon 12 with aspartic acid (G12D), which plays a crucial role in promoting tumor growth and spread (86).

A recent study demonstrated that engineered exosomes (iExosomes) could deliver specific siRNA or shRNA targeting the oncogenic KRASG12D. The delivery of these iExosomes significantly reduced levels of phosphorylated ERK protein and KRASG12D mRNA in human PANC-1 cells (87). During this process, CD47 present on exosomes protected them from phagocytosis by monocytes and macrophages, thus enhancing their efficacy (87). Although there have been relatively few clinical trials using exosomes as drug carriers, one such strategy is currently being employed in a phase I trial for PDAC patients with KRASG12D mutations (NCT 03608631). In this trial, mesenchymal stem cell-derived exosomes were used to carry siRNA targeting KRASG12D mutations in PC patients. In another study, CRISPR/Cas9 plasmid DNA was loaded into non-autologous exosomes which were then delivered to recipient cancer cells to induce targeted gene deletion (88). These CRISPR/Cas9-loaded exosomes specifically targeted the mutant KRASG12D oncogene allele in PCCs. By doing so, they inhibited the expression of the KRASG12D oncogene, thereby suppressing tumor growth both subcutaneously and in orthotopically modeled PC (62). Additionally, exosomes derived from hucMSCs were loaded with miR-145-5p and then administered to mice (46). These specific exosomes demonstrated anti-tumor properties by influencing the TGF-β/Smad3 pathway, inhibiting the proliferation and invasion of PCCs, and increasing apoptosis and cell cycle arrest (46). This research suggests that exosomes hold promise as effective drug delivery vehicles for gene therapy in PC.

Exosomes, with their unique capabilities and characteristics, have emerged as a highly promising system for the delivery of chemotherapeutic drugs. For instance, researchers have used sonication to load GEM into autologous exosomes, creating ExoGEM for targeted chemotherapy in PC (89). This study demonstrated that ExoGEM improved the release, delivery, cell absorption rate, and overall effectiveness of GEM treatment. Notably, ExoGEM significantly suppressed tumor growth and prolonged the survival of mice in a dose-dependent manner (89). Similarly, paclitaxel-loaded exosomes were found to be effective in inhibiting the proliferation of human pancreatic cell line CFPAC-1 owing to their potent anti-tumor activity (90). Furthermore, curcumin delivered through exosomes exerted cytotoxic effects within recipient PCCs *in vitro* (91).

Taken together, these findings suggest that exosome-based drug delivery systems can be used as promising vehicles for siRNA or inhibitors along with chemotherapeutic agents. This discovery opens up avenues toward developing safe and innovative therapeutic strategies for treating PC.

## 5 Limitation

However, there are still several limiting factors to consider in this article. Firstly, We only searched the WOSCC database. In order to ensure the integrity of the collected data, Scopus or Google Scholar and other databases can be used for comprehensive analysis in further studies. Secondly, this study only evaluates English articles or reviews, ignoring important studies in other languages or article types. Finally, we only selected articles published from January 1, 2013 to August 31, 2023, and it is possible to miss some important and landmark studies before 2013. In addition, due to the incomplete literature in 2023, some unpublished studies may be ignored, resulting in some new hotspots not being included. Nevertheless, literature analysis based on visualization can still effective help researchers understand the hotspots and emerging trends of exosomes for pancreatic cancer research. At present, the research on exosomes in PC mainly focuses on basic research. Therefore, attention should also be paid to translating fundamental research findings into clinical applications for the diagnosis and treatment of PC using exosomes.

## 6 Conclusion and future perspectives

This study shows that the role of exosomes in PC has drawn extensive scholarly attention, and this trend will continue in the future. The overall trend of publications shows annual increases, indicating that the interest of researchers in this field is growing. China has published the most papers in this field, while the US has the greatest influence, and China and the US work closely with other countries. The study also identified four different thematic clusters related to the role of exosomes in PC, including fundamental research on the basic mechanism of how exosomes act in PC as well as their potential as early diagnostic and prognostic markers for PC, clinical application in overcoming PC drug resistance, and potential utility as drug carriers. In summary, our study represents the first systematic bibliometric analysis of publications related to the role of exosomes in PC, providing an objective and comprehensive overview and valuable reference for researchers in this field.

PC, a complex and dangerous clinical condition, remains an ongoing challenge for clinicians and researchers. Although the serum biomarker CA 19-9 is commonly used in PC diagnosis, its sensitivity and specificity are significantly limited (55). The development of exosome-based drug carriers, therapeutic targets, and drugs for PC have shown encouraging results in preliminary experiments. Over the past decade, although substantial preclinical data related to the importance of exosomes have been reported, only a fraction of these findings have been directly translated into clinical trials. In a previous specific clinical trial (NCT 03608631, https://clinicaltrials.gov) KrasG12D siRNA delivery by exosomes has been performed as the first KRAS-targeted therapy. If successful, it could revolutionize the treatment for PC, marking a significant step toward the clinical application of exosomes in PC treatment (92). Exosomes could serve as ideal carriers for chemotherapeutic drugs such as paclitaxel (90), doxorubicin (93), and GEM (89). Another ongoing trial (NCT03821909) has assessed the diagnostic value of tumor cell-sourced exosomes circulating through the portal veins of patients with PC. Scientific advancements have provided methods for engineering exosomes; if technologies capable of carrying anticancer drugs can be successfully developed, utilizing exosomes for drug delivery could transform them into a preferred delivery system for cancer treatment (87, 94). Despite the potential for broad clinical application of exosomes, several challenges must be addressed before this can be achieved, including: (1) optimization of the techniques for separating and extracting large amounts of endogenous exosomes; (2) enhancement of delivery efficiency and targeting; and (3) assessment of the safety of exosomes as drug carriers. Resolving these problems requires further investigation, involving both preclinical studies alongside multicenter validation research, which necessitates extensive collaboration among researchers from various multidisciplinary fields.

## Author contributions

YjZ: Formal analysis, Investigation, Methodology, Software, Writing – original draft, Writing – review & editing. JF: Data curation, Writing – review & editing. QW: Formal analysis, Validation, Writing – review & editing. YZ: Data curation, Writing – review & editing. HD: Software, Visualization, Writing – review & editing. KJ: Software, Visualization, Writing – review & editing. HJ: Data curation, Supervision, Writing – review & editing. ZT: Data curation, Supervision, Writing – review & editing. RD: Conceptualization, Funding acquisition, Project administration, Resources, Supervision, Writing – review & editing, Writing – original draft.
