# Supplementary material for: Knowledge mapping and research trends of exosomes in pancreatic cancer: a bibliometric analysis and review (2013-2023)
Source: Front Oncol. 2024 Apr 24;14:1362436. doi: 10.3389/fonc.2024.1362436 (PMC11076735; doi:10.3389/fonc.2024.1362436)
Supplement: Supplementary file 1 [file DataSheet_1.docx]

Supplementary Material

**Knowledge Mapping and Research Trends of Exosomes in Pancreatic Cancer: A Bibliometric Analysis and Review (2013-2023)**

Yongjiang Zhou^1,2^, Jiajie Feng^1,2^, Qingqing Wang^1^, Yiwen Zhao^1,2^, Hanyu Ding^1,3^, Kexin Jiang^1,3^, Hua Ji^1,2^, Zheng Tang^1,2^, Ruiwu Dai^1,2,3,4*^

*** Correspondence:** Ruiwu Dai^*^: [dairuiwu@swjtu.edu.cn](mailto:dairuiwu@swjtu.edu.cn))

# Supplementary Data

# Supplemental File S1: ((“pancreatic cancer” OR “pancreatic carcinoma” OR “pancreatic neoplasm” OR “cancer of pancreas” OR “carcinoma of pancreas” OR “neoplasm of pancreas” OR “pancreatic ductal adenocarcinoma” OR PDAC) AND (exosomes OR exosome))
